# Lung Metastases of Pancreatic Adenocarcinoma Masquerading as Temporary Improvement of Bilateral Diffuse Lung Opacities Following Treatment for Interstitial Lung Disease

**DOI:** 10.1002/rcr2.70795

**Published:** 2026-10-22

**Authors:** Saki Kusunoki, Miki Tasato, Akihiko Sokai, Toshiyuki Iwata, Yasuyuki Hayashi, Naoaki Yasuda, Ikumi Kashiwagi, Takashi Nishimura

**Affiliations:** ^1^ Department of Respiratory Medicine Kyoto Katsura Hospital Kyoto Japan

**Keywords:** interstitial lung disease, lepidic growth pattern, lung metastasis, organizing pneumonia, pancreatic adenocarcinoma

## Abstract

A 75‐year‐old man presented with bilateral ground‐glass opacities (GGOs) and consolidation on computed tomography (CT). Bronchoalveolar lavage revealed no pathogens or malignant cells. Acute exacerbation of interstitial lung disease was suspected because of rapidly progressive respiratory failure, but steroid pulse therapy was ineffective. After cyclophosphamide treatment, CT showed transient improvement of GGOs and consolidation. Subsequently, worsening bilateral opacities and abnormalities of the main pancreatic duct were detected on CT and magnetic resonance imaging. These radiological findings and sputum cytology indicated lung metastases from pancreatic adenocarcinoma. The autopsy demonstrated lung metastases of pancreatic ductal carcinoma with extensive organizing pneumonia (OP). Although lung metastases from pancreatic cancer are occasional, diffuse bilateral opacities mimicking interstitial lung disease are rare. Improvement after prednisolone and cyclophosphamide may have reflected effects on both OP and metastases. Lung metastases should remain an important differential diagnosis even when bilateral GGOs and consolidation respond to treatment.

## Graphical abstract

A 75‐year‐old man was referred to our department with ground‐glass opacities (GGOs) and consolidation in both lungs on computed tomography. After an initial response to cyclophosphamide therapy, he returned with worsening dyspnea and was ultimately diagnosed with pulmonary metastases from pancreatic adenocarcinoma. This case highlights the importance of carefully considering when planning therapeutic strategies for undiagnosed lung disease.

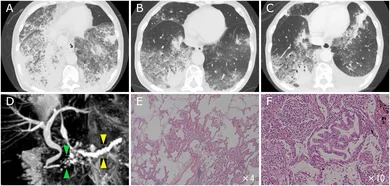

## 1 Introduction

Although lung metastasis of pancreatic adenocarcinoma is not uncommon, it may be mistaken for interstitial lung disease and lead to a delay in diagnosis when it presents as bilateral diffuse opacities. Furthermore, cyclophosphamide, which possesses both anti‐tumour and immunosuppressant effects, may yield therapeutic benefits for neoplasms and interstitial lung diseases, both of which are entirely distinct entities. Its pleiotropic effects are likely to further complicate diagnosis if treatments need to be started before diagnosis is made. We report a case in which lung metastases of pancreatic adenocarcinoma were diagnosed later, although we administered cyclophosphamide for interstitial lung disease, and the bilateral diffuse opacities improved temporarily.

## 2 Case Report

A 75‐year‐old man was referred to our department with ground‐glass opacities (GGOs) and consolidation in both lungs on computed tomography (CT) (Figure 1A). He was asymptomatic and had no history of smoking, dust, or other environmental exposure. His medication history was unremarkable. His oxygen saturation was 89% on room air. The C‐reactive protein and Krebs von den Lungen‐6 were 0.09 mg/dL and 225 U/mL, respectively, whereas serum Proteinase 3 anti‐neutrophil cytoplasmic antibody was 12.8 U/mL. No physical findings indicated an autoimmune disease. No symptoms of heart failure were observed and N‐terminal pro‐brain natriuretic peptide was 45 pg/mL. Carcinoembryonic antigen and soluble interleukin‐2 receptor were 2.0 ng/mL and 171 U/mL, respectively.

**FIGURE 1:**
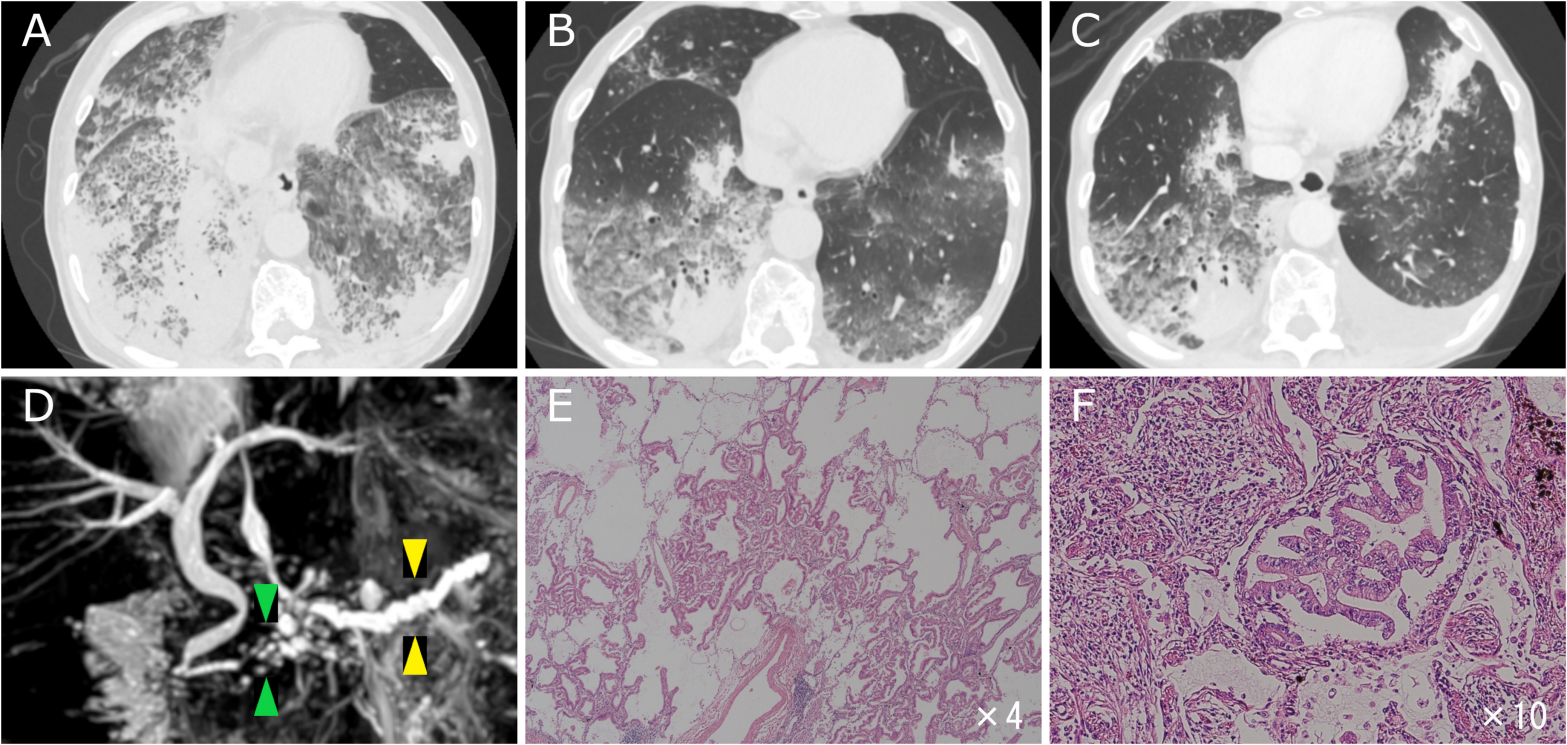
(A) Computed tomography (CT) depicting ground‐glass opacities (GGOs) and consolidation throughout both lungs. (B) CT following treatment with steroids and cyclophosphamide reveals a decrease in GGOs and consolidation in both lungs. (C) CT at the time of recurrent exacerbation revealed new consolidation and GGOs in both lungs, in addition to the pre‐existing lesions, along with a left pleural effusion. (D) Magnetic resonance imaging reveals dilation (yellow arrowheads) and disruption (green arrowheads) of the main pancreatic duct. (E and F) Pathological analysis reveals metastasis of pancreatic ductal carcinoma with a lepidic growth pattern in the lung (E; haematoxylin & eosin [H&E] staining, ×4), along with combined metastasis and organizing pneumonia (F; H&E staining, ×10).

Bronchoalveolar lavage (BAL) analysis was promptly performed and revealed neither pathogens nor malignant cells. The BAL fluid return was 41%, and the BAL fluid was slightly turbid and pale yellow. The total cell count was 3.0 × 10^5^ cells/mL, with a differential cell count of 87% macrophages, 11% lymphocytes, and 2% neutrophils. The CD4/CD8 ratio was 2.17. His oxygenation was impaired during the examination; therefore, a biopsy was not performed. Given the rapid worsening of his respiratory condition and radiologic findings following BAL, rapidly progressive interstitial lung disease of unknown aetiology was suspected. Methylprednisolone at 1000 mg/day for 3 days as steroid pulse therapy was administered. Despite steroid pulse therapy, his oxygenation status and radiological opacities did not improve. Therefore, two additional courses of methylprednisolone therapy were administered, followed by a reduction to oral prednisolone at 60 mg/day. At the time when the respiratory condition was the worst, high‐concentration oxygen with FiO_2_ of 100% was required via high‐flow nasal cannula. Intravenous cyclophosphamide therapy (500 mg/m^2^) was also administered every 2 weeks for a total of three doses, starting 3 weeks after the initial treatment. Prednisolone was subsequently tapered by 5–10 mg every 3–5 days. After administration of cyclophosphamide therapy, follow‐up CT showed a reduction in GGOs and consolidation in both lungs (Figure 1B). The patient was subsequently discharged 9 weeks after the initial treatment without oxygen therapy and with prednisolone of 20 mg/day.

Several days after the discharge, however, the patient presented to the emergency department with dyspnea. CT revealed no findings highly suggestive of malignancy, including lymphadenopathy or mass lesions, but new consolidation and GGOs in both lungs (Figure 1C). Magnetic resonance imaging revealed dilatation and disruption of the main pancreatic duct (Figure 1D). The levels of duodenal pancreatic cancer antigen 2, SPan‐1 antigen, and carbohydrate antigen 19–9 were 870 U/mL, 15 U/mL, and < 2 U/mL, respectively. Lung metastases from pancreatic adenocarcinoma were diagnosed based on radiological findings and sputum cytology that revealed adenocarcinoma. However, the patient was too debilitated and only received palliative care. The patient died, and an autopsy was performed. The autopsy revealed lung metastasis of pancreatic ductal carcinoma with a lepidic growth pattern, accompanied by abundant mucus production. Additionally, organizing pneumonia (OP) was widely present in both lungs (Figure 1E,F).

## 3 Discussion

Lung metastases of pancreatic cancer are reported in 15%–50% of cases and are not uncommon [1]. CT features of lung metastases in patients with pancreatic cancer include various patterns of nodules, GGOs, and consolidation [1]. Alveolar patterns, including GGOs and consolidation, suggest the presence of a lepidic growth pattern in lung metastases of pancreatic cancer [1]. Lung metastases of pancreatic cancer typically present as isolated, asymmetrical, or multifocal lesions, whereas they may rarely present as bilateral diffuse lung opacities that mimic interstitial lung disease [2].

Interstitial lung diseases and neoplasms are sometimes related. It is well‐known that cancer can develop from fibrotic interstitial lung disease, but cancer can also cause various patterns of interstitial lung disease. Carcinomatous lymphangiopathy is known as a diffuse lung opacity closely associated with carcinoma. However, in the present case, pathological findings on autopsy did not reveal carcinomatous lymphangiopathy. Instead, pancreatic ductal carcinoma and OP were identified. The development of interstitial lung diseases, including OP due to treatments including chemotherapy and immunotherapy, is well‐known. Moreover, OP may also occur in association with the cancer itself. OP has been reported to complicate not only lung cancer but also other malignant tumours such as leukaemia and gastrointestinal cancers [3]. Although several studies have suggested that secondary OP, including tumour‐related OP, and cryptogenic OP may have similar treatment responses [4], the evidence remains limited by the small number of cases of tumour‐related OP; therefore, definitive conclusions cannot be drawn.

Although cyclophosphamide is not commonly used in the current standard chemotherapy regimen for advanced pancreatic cancer, a study investigating its role as maintenance therapy for pancreatic adenocarcinoma revealed that it may be effective as a standalone agent [5]. In the present case, despite the underlying malignant disease, temporary improvement followed treatment for interstitial lung disease. The temporary improvement in pulmonary opacities may have resulted from prednisolone and/or cyclophosphamide, which were effective against OP and possibly lung metastases, although distinguishing between these entities was challenging.

This case highlights that even when diffuse bilateral GGOs and consolidation respond to treatment for interstitial lung disease, lung metastasis from extrapulmonary malignancies remains a critical differential diagnosis and should be carefully considered when planning therapeutic strategies for undiagnosed lung disease.

## Author Contributions

**Saki Kusunoki:** writing – original draft, visualization. **Miki Tasato:** supervision. **Akihiko Sokai:** supervision, writing – original draft, writing – review and editing. **Toshiyuki Iwata:** supervision, writing – review and editing. **Yasuyuki Hayashi:** supervision. **Naoaki Yasuda:** supervision. **Ikumi Kashiwagi:** supervision. **Takashi Nishimura:** supervision.

## Funding

The authors have nothing to report.

## Consent

The authors declare that written informed consent was obtained from the patient's family for the publication of this manuscript and accompanying images using the consent form provided by the Journal.

## Conflicts of Interest

The authors declare no conflicts of interest.

## Data Availability

The data that support the findings of this study are available from the corresponding author upon reasonable request.
